# Sunlight Effects on Immune System: Is There Something Else in addition to UV-Induced Immunosuppression?

**DOI:** 10.1155/2016/1934518

**Published:** 2016-12-13

**Authors:** D. H. González Maglio, M. L. Paz, J. Leoni

**Affiliations:** Instituto de Estudios de la Inmunidad Humoral (IDEHU), CONICET, Universidad de Buenos Aires, Junín 956, C1113AAD Buenos Aires, Argentina

## Abstract

Sunlight, composed of different types of radiation, including ultraviolet wavelengths, is an essential source of light and warmth for life on earth but has strong negative effects on human health, such as promoting the malignant transformation of skin cells and suppressing the ability of the human immune system to efficiently detect and attack malignant cells. UV-induced immunosuppression has been extensively studied since it was first described by Dr. Kripke and Dr. Fisher in the late 1970s. However, skin exposure to sunlight has not only this and other unfavorable effects, for example, mutagenesis and carcinogenesis, but also a positive one: the induction of Vitamin D synthesis, which performs several roles within the immune system in addition to favoring bone homeostasis. The impact of low levels of UV exposure on the immune system has not been fully reported yet, but it bears interesting differences with the suppressive effect of high levels of UV radiation, as shown by some recent studies. The aim of this article is to put some ideas in perspective and pose some questions within the field of photoimmunology based on established and new information, which may lead to new experimental approaches and, eventually, to a better understanding of the effects of sunlight on the human immune system.

## 1. Introduction

Sunlight is composed of ultraviolet (UV), visible, and infrared radiations. It is essential for life on earth as a source of energy, light, and warmth and to maintain oxygen levels in our atmosphere, due to the role it plays in photosynthesis. However, it also causes profound changes in the human body.

The effects of sunlight, particularly UV radiation, on the skin cell biology as well as on the immune system have been described at length. One of its most important effects is UV-induced immunosuppression, a defective immune response triggered by UV radiation affecting the skin first, and then the whole body. Thousands of experimental papers have been published since the first descriptions of UV-induced immunosuppression and its role in the development of skin carcinogenesis [1–4]. In addition to causing immune cell alterations, UV radiation absorption produces molecular changes, many of which have been extensively reported (though it is impossible to know if all types have been covered). UV radiation is known to be directly absorbed by DNA (in particular by adjacent pyrimidine bases) and by cis-urocanic acid in exposed cells [5–7] and to promote the production of reactive oxygen species (ROS), which in turn may cause DNA damage [8]. These alterations lead to changes in the production of different molecules related to the immune system, including interleukin-10 (IL-10), IL-4, and prostaglandin E_2_ (PGE_2_) [9–11]. These molecules, in turn, modulate systemic immune responses, promoting defects in cellular immunity [12–14]. In animal models, it has been demonstrated that UV-induced systemic immunosuppression is related to the development of antigen-specific regulatory T-cells (CD4+ CD25+ foxp3+ cells), which can be transferred into nonexposed animals [15, 16]. The development of these regulatory cells is associated with a particular environment of soluble molecules established after UV exposure, which include not only cytokines and PGE_2_ but also Vitamin D (its role in UV-induced immunosuppression will be discussed below) [17]. It is known that this environment may condition skin dendritic cells in order to specifically promote the regulatory T-cell phenotype during priming in regional lymph nodes [18, 19]. The induction of the tolerogenic phenotype in dendritic cells may be so intense that even bone marrow cells may develop it, leading to suppressive responses several days (and even months) after exposure [20]. However, regulatory T-cells and tolerogenic dendritic cells are not the only ones involved in UV-induced immunosuppression. Mast cells also have a role to play in the development of immunosuppression, since the number of such cells in the skin and their migration to draining lymph nodes have been correlated with the UV-induced suppressive response [21, 22]. Moreover, regulatory B-cells, capable of affecting dendritic cell-mediated T-cell activation, are also involved in this effect triggered by UV exposure. Their number and suppressive action in draining lymph nodes increase after UV exposure [23]. Molecular mechanisms involved in this effect include the production of IL-10 by regulatory B-cells after the interaction of the platelet-activation factor, a proinflammatory mediator, with its receptor in B-cells [24]. Finally, oxidative stress is also related to UV-induced immunosuppression, since the topical application of antioxidants before UV exposure may completely inhibit it [25].

Regardless of the cell types involved, an important biological consequence of the UV-induced immunosuppression is the loss of immunosurveillance on newly generated malignant cells. Skin cell DNA is affected by UV radiation either directly (dimerization of adjacent pyrimidines) or indirectly (oxidative damage induced by ROS), which may cause specific mutations that will eventually lead to the malignant transformation of these cells (mainly, melanocytes and keratinocytes) [26–28]. These malignant cells, under normal circumstances, can be identified and eliminated by the immune system in a process known as “immunosurveillance.” However, after only a single exposure to UV radiation, this immune process can be severely affected, diminishing the ability of the body to fight skin tumors.

But sunlight exposure is not only associated with detrimental effects on human health. Sunlight exposure is essential to ensuring proper levels of circulating Vitamin D, since its synthesis is initiated in the skin with the photoconversion of 7-dehydrocholesterol to previtamin D [29]. Vitamin D is essential to maintain bone homeostasis, but it also has effects on the immune system [30, 31]. The role of Vitamin D in UV-induced effects will be discussed below, but it is worth noticing that this is one of the main benefits of sunlight exposure.

The above-mentioned effects of sunlight, especially UV radiation, on human health have been also widely reviewed. Plenty of excellent reviews covering the contrasting consequences of skin exposure to sunlight have been published [17, 32–42]. This article is intended to raise questions regarding the overall effects of skin exposure to sunlight that may lead to the use of different sources of radiation in the treatment of human diseases.

## 2. How Low Is a Low-Immunosuppressive-UV Dose?

The pioneering work of Dr. Kripke demonstrating UV-induced immunosuppression was based on chronic exposures to UV radiation (three times a week during three months) [1, 2]. Since then, a great number of irradiation protocols inducing immunosuppression were created.  Table 1 briefly summarizes some of the doses used in such protocols with their respective correlation, in most of the cases, to a biological effect: the minimal erythema dose (MED).

It should be noted that the paradigm of UV-induced immunosuppression has changed over the last decades. While immunosuppression in Dr. Kripke's work was reached by chronic irradiation, later on it was proved that a single high irradiation (above the erythema dose) was also capable of producing the same effect. Regardless of the form of irradiation involved, some of the papers mentioned in Table 1 refer to the source of irradiation as a “low-dose UV” [46, 51, 53–55] that can promote immunosuppression even under the MED. Consequently, the concept of UV dose causing immune suppression was then correlated to human exposures to sunlight, alerting about exposures even below the minimal erythema dose. Indeed, many works have demonstrated that immunosuppressive doses of UV radiation in humans are effectively below the MED. Wolf et al. published that the dose capable of producing 50% of the inhibition of CHS response to DNCB during sensitization phase ranged between 0.63 and 0.79 MED [56]. It is worth noticing that a major effect of sunlight on human health, as reviewed, is UV-induced immunosuppression, which is presented as having both a positive and a negative effect on human health. An example of this is the review by Dr. Schwarz [40]. Negative effects are related to defective immunosurveillance, allowing for tumor development, while the positive effects are related to the control of autoimmune diseases due to the generation of specific regulatory cells. In the latter case, the possible implications of the above-mentioned “low UV doses” were reviewed a few years ago by Dr. Halliday et al. [32], which led to the design of clinical trials to assess the role of phototherapy in autoimmune diseases [57].

However, our question addresses the fact that half MED is not as low as a tenth (or even less) of the MED, which we classify as “very low doses.” These “very low doses” of radiation are completely relevant if we think in photoprotectors not as blockers of radiation but as filters: radiation can be absorbed to a high degree (SPF 50 or more) but not entirely. Thus, SPF (Sun Protection Factor) is “a measure of how much solar energy (UV radiation) is required to produce sunburn on protected skin (i.e., in the presence of sunscreen) relative to the amount of solar energy required to produce sunburn on unprotected skin,” according to the U.S. Food and Drug Administration [58]. However, it has been stated that the SPF of a given sunscreen may not be directly related to its Immune Protective Factor (IPF) [59], leading to the need of a standardized procedure to evaluate IPF. This topic is remarkably well treated in a publication of five groups of researchers from Australia, Austria, France, UK, and USA [60]. Based on SPF definition, we can estimate the approximate time of exposure to receive a tenth of the MED using SPF 50. For example, as it was published by Samanek et al., in Sydney (Australia) in summer time, people with skin phototype II required an exposure of 11 minutes to reach MED [61]. In that context and using a SPF 50 photoprotector, a tenth of the MED would be achieved after a 55-minute exposure. Moreover, these doses of radiation can also be obtained while walking normally outdoors in daylight in summer. One is likely to be frequently exposed to the above-mentioned “very low doses” of UV, but will such doses also cause an immunosuppressive effect? Or will they produce other effects on human health? These questions, which have been recently raised, will be discussed in another section.

## 3. Is Vitamin D a Soluble Mediator of UV Radiation Immune Effects or Just an Epiphenomenon?

It is very well known that UV radiation is essential for Vitamin D synthesis, in particular for the photoconversion of 7-dehydrocholesterol to cholecalciferol in the epidermis. The production of this vitamin represents one of the most important beneficial effects of sunlight exposure. Vitamin D synthesis and its impact on human health have been extensively reviewed in many of the cited papers [31, 36, 62–65] and it is not our purpose to analyze once again the relevance of this process.

However, there is some puzzling evidence of the role of Vitamin D in UV-induced immunosuppression: (a) Vitamin D is a mediator of UV-induced immunosuppression and it mimics this effect [46]; (b) Vitamin D is not necessary to immunosuppress UV irradiated animals [46, 47]; (c) Vitamin D and a nongenomic analog are protective against UV-induced immunosuppression and carcinogenesis [52]. Do these differences lie on the concentration used and/or particular preactivated pathways? Schwarz et al. [46] topically applied 0.1 *μ*g of 1,25(OH)_2_VitD_3_ (diluted in acetone/olive oil, 4 : 1), which represents 240 pmoles, in order to induce immunosuppression. On the other hand, Dixon et al. [52] used 159.6 and 44.8 pmoles (diluted in ethanol, propylene glycol, and water to a final solvent ratio of 2 : 1 : 1, resp.) of the vitamin in order to obtain significant protection against UV-induced immunosuppression. Even though the concentration of Vitamin D used in these experiences is different, an important question arises: which one best represents the concentration of vitamin in the skin after UV exposure? Gorman et al. measured Vitamin D in the ear skin of irradiated animals (on a Vitamin D replete or deficient diet) and they reported that “no change in 1.25(OH)_2_D3 levels was detected in the ear skin of either male or female Vitamin D3-deficient mice with UV irradiation” [47]. It is difficult to find out the role of this vitamin in UV-induced immunosuppression in humans, but we would like to discuss two papers related to human exposure to radiation. In a study with volunteers subjected to a CHS protocol, a single exposure to 3 MED (an average dose of 420 mJ/cm^2^, since MED depends on skin type) prior to sensitization significantly suppressed the immunological reaction [66]. On the other hand, measurements performed in a group of Danish people during a summer holiday revealed that with a total exposure of 10100 mJ/cm^2^ (an average for 25 individuals) serum concentration of Vitamin D increased only 1.44 times [67]. It seems like biological Vitamin D increments after UV exposure are not sufficient to justify the suppression of specific immune responses, but certainly more evidence is needed.

Finally, to conclude this topic, there is a very good review by Dr. Byrne, which we completely agree with, that presents a conclusion about a clinical trial on the use of Vitamin D in autoimmune diseases suggesting that “boosting Vitamin D levels alone may not have the desired therapeutic effect and that there is something else about UV exposure that explains the protective properties of sunlight” [42].

## 4. What Determines Different Susceptibility to UV-Induced Immunosuppression in Mice? Is There a Correlation in Humans?

Susceptibility to UV-induced immunosuppression is very well known. Yoshikawa et al. used CHS reaction in humans to study this phenomenon [68]. They observed an absence of immunosuppression in 60% of the healthy volunteers analyzed. In contrast, patients with a history of nonmelanoma skin cancer showed marked susceptibility to immunosuppression. They posed the possibility that this increased susceptibility may be the cause of skin cancer development. Moreover, twenty years ago, Noonan and Hoffman performed an extensive analysis of susceptibility to CHS reactions in 16 strains of inbred mice [69]. They informed the UV radiation dose needed to produce a 50% inhibitory effect on a CHS protocol, which ranged from 70 mJ/cm^2^ to 260 mJ/cm^2^ in highly susceptible mice (C57 mice); from 470 mJ/cm^2^ to 690 mJ/cm^2^ in moderately susceptible mice (DBA/2 and A/J mice); and from 930 mJ/cm^2^ to 1230 mJ/cm^2^ in low susceptible mice (Balb/c mice). This almost 20-time difference in the UV doses causing immunosuppression reflects substantial differences in the genetic background involved in the response to UV light. Hair pigmentation does not seem to justify these results, since C57 (highly pigmented mice) are more susceptible than Balb/c (albino nonpigmented mice). What differences in what genes can condition UV radiation response? In a more recent work, Welsh et al. investigated the association of human polymorphisms in functional variants of 10 genes involved in the response to UV radiation (IL-10, IL-4, and TNF-*α* among others), with the risk of Basal Cell Carcinoma and Squamous Cell Carcinoma (both associated with UV-induced immunosuppression) [70]. Major effects were observed for skin type, lifetime severe sunburns, and IL-10 haplotypes in both BCC and SCC. The haplotypes studied were in the promoter region of the gene and may be correlated with an increased production of this cytokine [71]. Moreover, Nagano et al. published a study on a non-Caucasian population, where similar results were observed: patients who developed skin cancer in sun-exposed areas (more susceptible to immunosuppression) presented less frequency of low expression IL-10 promoter genotype [72]. The work by Welsh and collaborators reinforces the idea of the impact of UV exposure in the development of skin cancer and UV-induced immunosuppression, since skin type and burns were highly associated with the development of tumors, but it also incorporates the notion of genetic susceptibility, as it was demonstrated long ago by Yoshikawa, in humans, and Noonan and Hoffman, in mice.

We have recently studied the response of different mice strains to a single, 2 MED, UVB exposure and observed that C57BL/6 mice greatly differ in their inflammatory and oxidative responses from Balb/c and other mice strains. C57BL/6 mice produce a stronger inflammatory response (increased levels of serum and epidermal IL-6) and a weaker oxidative epidermal response (with less production of superoxide anion in epidermal cells) after irradiation [73].

Even though differences in the response to UV light between individuals with different genetic background exist, we cannot precisely determine their influence on the overall response to natural or therapeutic exposures. In the future, this issue may be unraveled, once more studies are performed in the area.

## 5. Is There Something Else (with Immune Effects) in Sunlight than UV Radiation?

Sunlight is a complex source of different types of radiation that includes UV radiation but it also exceeds it. The impact of a specific source of radiation on living organisms depends on the capacity of the cells to absorb it by producing molecules with a specific absorption spectrum. Once radiation is absorbed by these molecules, different cellular pathways can be activated. In this way, UV radiation is absorbed by DNA, cis-urocanic acid, and proteins, among other molecules, and promotes many of the very well-known effects mentioned before. But, are visible radiations absorbed by skin cells? Or in other words, do visible radiations directly induce any effect, either positive or negative, on skin health? The answer is yes. Laser therapies with visible wavelengths for cosmetic or therapeutic purposes are distributed worldwide, but do they only promote beneficial effects? What cellular pathway do they affect?

In order to analyze some specific responses activated by visible light, there is an increasing number of reports describing different effects of these radiations on skin or its constituent cells. It has been published that low level light therapy with different wavelengths (470 nm, blue, and 629 nm, red) induces angiogenesis and improves wound healing in an ischemia disturbed rodent flap model, supporting an interesting application of artificial light sources on human health [74]. Another benefit is a specific bactericidal effect on* Staphylococcus aureus *and* Pseudomona aeruginosa*, which has been proved both in vitro (in bacterial culture techniques) and in vivo (in infection models) [75, 76]. The last reference is a work by Dai et al. which used blue light (415 nm) therapy to effectively treat a potentially fatal mice infection with* Pseudomona*, showing that the bactericidal effect could be very useful in skin infections, especially in those produced by multiresistant microorganisms. Therefore, the questions that arise are the following: are both (healing and bactericidal) effects simultaneously produced? Is it possible for blue light to modulate immune responses? These questions need to be addressed in new experimental studies in order to test, for example, blue light therapy in different skin conditions in which healing and antimicrobial barriers have to be rapidly improved, like in massive burns.

However, blue light has also shown some potentially harmful effects. As an example, Mamalis et al. described that this type of radiation promotes a decrease in skin fibroblasts proliferation as well as an increase in ROS production [77]. ROS and matrix degrading enzymes production increase is a very well-known harmful effect of UV radiation, but this is also caused by visible light [78]. In addition, a recently published article by Vandersee and collaborators demonstrated that blue light skin exposure affects the antioxidant defense of the skin, by decreasing the cutaneous carotenoid concentration, in addition to its effects as a promoter of ROS, as previously described [79]. Does this blue light-induced oxidative damage also affect keratinocytes and Langerhans cells? If so, how does it affect the immune functions of those cells?

We would like to briefly comment on another skin cellular response to radiation: the induction of pigmentation. This important response of the skin to sunlight is thought to have a protective role against DNA damage [80]. It has been proved that blue light (415 nm), but not red light (630 nm), is able to induce pigmentation in type III and IV healthy subjects. Moreover, when compared to UVB irradiation, the blue light induces a significantly more pronounced hyperpigmentation that lasts up to 3 months [81]. This means that blue light is capable of affecting epidermal cells and, in particular, that melanocytes are very sensitive to this radiation. Again, it raises the question about blue light effects on skin immune cells. There are just a few reports about the effects of blue light on dendritic or Langerhans cells. On the one side, it has been shown that during a photodynamic therapy with blue light the number of epidermal Langerhans cells was not affected; neither was the oxidative damage to their DNA, by the radiation itself or the photosensitizer [82, 83]. But on the other side, in vitro irradiation of dendritic cells affects their capacity to respond to a LPS/IFN-*γ* stimulus, thus decreasing the production of cytokines (IL-12, IL-6, and TNF-*α*) and the level of expression of costimulatory molecules (CD83 and CD80) in a dose dependent fashion [84, 85].

**Figure 1:**
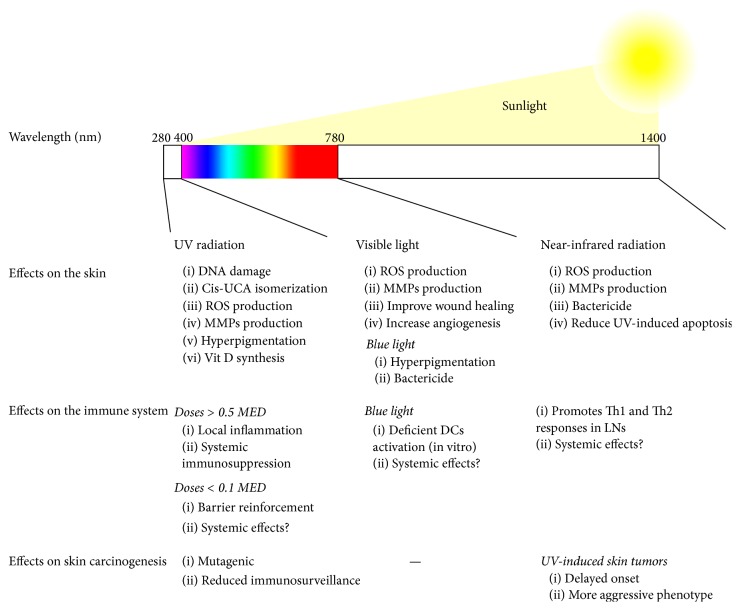
Sunlight effects on the skin and the immune system. The effects triggered by different wavebands of radiation are summarized, focusing on those effects described in the text. UCA: urocanic acid; ROS: reactive oxygen species; MMPs: matrix metalloproteases; MED: minimal erythema dose; DCs: dendritic cells; LNs: lymph nodes.

The exact role of blue light in immunosuppression and the possible involvement of visible radiations in the very well-known detrimental effects of sun exposures are yet to be explored. But sunlight includes much more than UV and visible light. Infrared- (IR-) A radiation (780–1400 nm), which accounts for more than 30% of sunlight, can penetrate deeply into the skin and promote the production of ROS and MMP-1 by skin cells [86–88]. Moreover, IR exposure may have other effects on skin cells, modulating their response to deleterious doses of UV radiation. Jantschitsch et al. showed that IR-exposed keratinocytes were protected from UV-induced apoptosis, through the reduction of DNA damage and modulation of the expression of apoptosis-related proteins (upregulation of antiapoptotic FLIP_L_ and BCL-X_L_ and downregulation of proapoptotic BAX) [89]. These cellular alterations lead to a delay in the onset of skin cancer development in an in vivo model but promote a more aggressive phenotype of the tumors developed [90]. Regarding IR effects on the immune system, Lee et al. have recently published that IR exposure promoted an increase in the number of epidermal Langerhans cells, while lymph nodes cells stimulated with an anti-CD3 antibody led to the production of Th1 and Th2 cytokines, but not to regulatory ones [91]. These effects may be used to modulate immune responses locally and systemically. The IR/far red light mediated photobiomodulation has been described as an effective treatment for cutaneous infection by methicillin-resistant* Staphylococcus aureus* [92, 93], experimental autoimmune encephalitis (a mouse model of multiple sclerosis) [94], and brain disorders in humans [95]. The contribution of these wavebands to the final effects of sunlight exposure remains to be elucidated.  Figure 1 summarizes the effects triggered by different wavebands of sunlight on the skin and the immune system.

## 6. Are “Very Low UV Doses” Free of Immunomodulatory Effects?

To conclude this review, we would like to return to the discussion about the “very low UV doses” mentioned above. These doses of radiation we all tend to be exposed to—during occasional walks under the sun in summer time or during intentional sun exposures using sun protectors—are very relevant to study and we are working in this direction in our laboratory. We have recently published an article in which we worked with the idea of a model of daily “casual” exposures to sunlight, in particular to UV radiation. We performed UVB irradiation of hairless mice during four consecutive days with only 20 mJ/cm^2^ (a tenth of the MED, described as repetitive low UV doses or rlUVd) and compared the effects on skin innate immunity with animals exposed to a single high dose irradiation (400 mJ/cm^2^, 2 MED, described as single high UV dose or shUVd). We found a strong inflammatory response, as it has been largely described, in shUVd exposed animals which was completely absent in the rlUVd exposed ones. However, these “very low-dose” irradiations are far from producing no alterations at all, since a strong reinforcement of the epidermal barrier function was observed in mice irradiated with rlUVd. This reinforcement is based in a slight increment in epidermal thickness, without signs of histological alteration or metabolic dysfunction of epidermal cells, and a strong induction of antimicrobial peptides transcription [96]. The reinforcement of the barrier function was also described by Hong et al., who reported an increase in antimicrobial peptides' synthesis and permeability barrier reinforcement (measured after a tape-stripping insult) in hairless mice exposed to 40 mJ/cm^2^ just once or three times in consecutive days [97].

Does this type of irradiation impact only innate skin immunity? It is difficult to find evidences of adaptive immunity-based reinforcement by repetitive low UV doses. However, more than a decade ago, Khaskely et al. published a very interesting work on cutaneous leishmaniasis in mice model (using Balb/c mice) [98], in which an experimental infection with parasite* Leishmania amazonensis* was introduced after a UVB irradiation procedure that consisted of daily exposures to 25 mJ/cm^2^ during four consecutive days (very similar to the schedule used in our lab). Twenty-four hours after the last exposure, mice were challenged intradermally with the parasite. They found that the development of skin lesion produced by the infection was significantly reduced by a pretreatment with low-dose UV irradiation. The authors also reported an increase in serum IFN-*γ* as a finding that could explain the control of the infection, since* L. amazonensis* is an intracellular parasite that is capable of surviving within the macrophages. This essential article raises new questions again: are repetitive low UV doses capable of predisposing adaptive immunity to a stronger response? Or does it only promote a better response of skin macrophages without T-cell activation? Is it possible to obtain similar responses in immune reinforcement with rlUVd once the infection has developed?

Unfortunately, neither the authors nor other researchers have published any new articles on this topic since then, but the questions remain there to be investigated and we ourselves are trying to answer some of them.

## 7. Conclusions

Sunlight exposure cannot be considered only as a carcinogen nowadays, though the highly relevant mechanisms leading to immunosuppression and consequently to skin cancer development have been and are still very well characterized and the most reported in the literature. There is also abundant evidence showing that sunlight effects, especially of “very low doses,” are indeed beneficial and not only due to Vitamin D synthesis. We believe that plenty of work has yet to be done in the field of photoimmunology, which needs to cover the impact not only of “very low doses” of radiation, but also of exposure to non-UV light (focusing on the effects of various doses) on the immune system. We as immunologists and particularly photoimmunologists have to move forward from the milestone of UV-induced immunosuppression towards a more comprehensive analysis of the interaction of human beings with the environment, leading to the possibility of establishing new therapies, which might be useful in different pathologies and not only in those that require a specific suppression of the immune response.

## Figures and Tables

**Table 1:** Summary of irradiation protocols producing UV-induced immunosuppression. The experimental design of all the papers cited in the table included the irradiation before the sensitization stage of the immune responses. CHS: contact hypersensitivity; DTH: delayed type hypersensitivity; Oxa: oxazolone; DNFB: 2,4-dinitrofluorobenzene; Ova: ovalbumin.

Authors	UV source	UV dose employed	MED represented	Mice strain	Type of reaction (antigen)
Reeve et al. [43]	UVA/UVB	3 × 4500 mJ/cm^2^ (UVA); 3 × 252 mJ/cm^2^ (UVB)	1 MED	C57BL/6	CHS (Oxa)
Majewski et al. [44]	UVR	4 × 150 mJ/cm^2^	Not expressed	C57BL/6	CHS (DNFB)
Wang et al. [45]	UVB	3 × 45 mJ/cm^2^	Not expressed	C57BL/6	CHS (DNFB) OT1 T-cell proliferation (Ova)
Schwarz et al. [46]	UVB	4 × 150 mJ/cm^2^	Not expressed	C57BL/6	CHS (DNFB)
Gorman et al. [47]	65% UVB	400 and 800 mJ/cm^2^	>1 MED	Balb/c and C57BL/6	CHS (DNFB)
Zhang et al. [48]	45% UVB	750 mJ/cm^2^	Not expressed	C57BL/6	CHS (DNFB)
Guéniche et al. [49]	UVB + UVA	Not expressed	2.5 MED	SKH:HR1	CHS (DNFB)
Shreedhar et al. [9]	65% UVB	1500 mJ/cm^2^	Not expressed	C3H/HeN	DTH (alloantigen)
Li et al. [50]	UVB	200 mJ/cm^2^	Not expressed	C57BL/6	CHS (DNFB)
Rana et al. [51]	UVB	3 × 150 mJ/cm^2^	0.5 MED	C57BL/6	DTH (Ova)
Dixon et al. [52]	UVB and UVA	3 × 400 mJ/cm^2^ (UVB)	3 MED	SKH:HR1	CHS (Oxa)
